# Water Sorption, Solubility, and Translucency of 3D-Printed Denture Base Resins

**DOI:** 10.3390/dj10030042

**Published:** 2022-03-09

**Authors:** Mohammed M. Gad, Saleh Z. Alshehri, Shahad A. Alhamid, Alanoud Albarrak, Soban Q. Khan, Faris A. Alshahrani, Firas K. Alqarawi

**Affiliations:** 1 Department of Substitutive Dental Sciences, College of Dentistry, Imam Abdulrahman Bin Faisal University, P.O. Box 1982, Dammam 31441, Saudi Arabia; faalshahrani@iau.edu.sa (F.A.A.); fkalqarawi@iau.edu.sa (F.K.A.); 2 College of Dentistry, Imam Abdulrahman Bin Faisal University, P.O. Box 1982, Dammam 31441, Saudi Arabia; drsalehalshehri19@gmail.com (S.Z.A.); 2160001939@iau.edu.sa (S.A.A.); 2160001708@iau.edu.sa (A.A.); 3 Department of Dental Education, College of Dentistry, Imam Abdulrahman Bin Faisal University, P.O. Box 1982, Dammam 31441, Saudi Arabia; sqkhan@iau.edu.sa

**Keywords:** denture base resin, translucency, three-dimensional printing, water sorption, solubility

## Abstract

This study aimed to evaluate the water sorption, solubility, and translucency of 3D-printed denture base resins (NextDent, FormLabs, and Asiga), compare them to heat-polymerized acrylic denture base resins, and assess their performance under the effects of thermal cycling. A total of 80 acrylic disc specimens were used in the current study, categorized into four groups (*n* = 10); in one group, the samples were fabricated conventionally with a heat-polymerizing process (control), while the other three groups were fabricated digitally from different 3D-printed reins (NextDent, FormLabs, and Asiga). Specimens were fabricated according to the manufacturers’ recommendations and immersed in distilled water for 48 h at 37 °C. Data on water sorption, solubility, and translucency measurements (T1) were obtained. All the specimens were subjected to 5000 thermal cycles, and then the measures were repeated using the same method (T2). Data analysis was attained via ANOVA and the post hoc Tukey test (α = 0.05). The type of resin significantly affected the values of water sorption, solubility, and translucency (*p* < 0.001). The water sorption of 3D-printed resins was increased significantly in comparison to control with or without a thermal cycling effect. In terms of solubility, a significant increase in 3D-printed resins before thermocycling was observed; however, after thermocycling, Asiga had a significantly low value compared to the other groups (*p* < 0.001). Thermal cycling increased the water sorption and solubility of all tested materials. In comparison to control, the translucency of the 3D-printed resins was significantly decreased (*p* < 0.001). The translucency was significantly decreased per material in terms of the thermal cycling effect (before and after). NextDent showed significantly low translucency values (*p* < 0.001) compared to the other groups. All 3D-printed resin groups had higher water sorption and solubility and lower translucency values in comparison to the heat-polymerized resin group. Regardless of resin types, thermal cycling adversely affected all tested properties.

## 1. Introduction

Complete dentures still remain a satisfactory treatment modality for edentulous patients, especially those who cannot afford implant-supported fixed dental prosthesis. It is well known that the conventional denture fabrication method entails a tedious and labor-intensive laboratory process; digital systems have been developed to improve the production of denture bases and eliminate the common disadvantages related to the traditional denture fabrication process [1].

As an alternative to conventional methods, computer-aided-design/computer-aided-manufacture (CAD/CAM) technology was introduced to fabricate dentures digitally. Digital denture fabrication was introduced first as a subtractive method where the dentures were milled from prefabricated resin block. Later on, the additive method was developed, where dentures were digitally fabricated layer by layer via three-dimensional printing technology (3D-printing), using photo-polymerized fluid resins [2,3]. CAD/CAM dentures are fabricated without any labor-intensive procedures and in a timely manner in comparison to previous conventional methods, in addition to the ability to duplicate the existing dentures with enhanced adaptation to the underlying tissues [2,4]. Additionally, this positively impacts the patients’ experience by reducing the clinical chair-side time, since fewer laboratory procedures are involved, which ultimately reduces the fabrication errors accompanied by the conventional method [2,3].

Ever since digital dentistry has been introduced, the subtractive method has been the most popular method; however, the additive method has overcome a few of the subtractive method’s shortcomings. The 3D-printing method, an additive method, exhibits some advantages and is considered economical compared to the subtractive method [1,5]. The 3D-printed dentures are fabricated with an accurate measurement of the fluid resin needed, and they are made in an appropriate time without waste material [5,6]. However, CAD/CAM-milled resins prevail when it comes to clinical implications; 3D-printed resins had issues due to their low performance [7]. Studies have reported that 3D-printed resin had flexural and impact strength values comparable to those of conventional fabricated resins, and they were clinically acceptable [3]; however, further investigations were needed to evaluate their overall mechanical properties and test how they would perform after being exposed to different treatment modalities.

One of the properties of acrylic resins is water sorption, and this affinity to water alters its physical properties and results in dimensional changes in the denture base, which cause internal stresses that adversely impact the denture’s long-term success [8,9]. It is important to ensure that the material’s water sorption/solubility properties are kept at a minimum in order to prevent fractures and cracks, thus improving the denture’s stability [10]. There are three types of materials which are soluble in denture base resins: initiators, plasticizers, and free monomers [1,11]. Residual monomers have been linked to increased water sorption/solubility, consequently leading to dimensional instability [12]. Water solubility is an undesirable property, since more unreacted resin will exist; hence, unreacted monomers can have a negative influence on the oral tissue and can increase the tissue reaction. Therefore, prostheses are to be compatible and insoluble in patients’ mouths [12,13].

Recent studies have shed light on 3D-printed resins and their application in dentistry, with a major focus on the mechanical behavior of 3D-printed resins [1,5,9] to overcome the limitation of its clinical use. However, the physical properties of 3D-printed resins and their performance under different tests are yet to be investigated thoroughly. Berli et al. investigated the water sorption/solubility of 3D-printed acrylic resins for occlusal devices; their report indicated that 3D-printed resins had higher water sorption/solubility values than other resins [9].

The aesthetics of removable prostheses is considered an important factor in meeting patients’ expectations. Therefore, ensuring that the denture base’s color matches the color of underlying soft tissues is an essential requirement for the overall aesthetics of dentures [14,15,16]. The translucency of removable prostheses plays a major role in the aesthetics of denture base acrylic resins [14]. Translucency is defined as the ability of the material to permit light to pass through its structure, allowing the background to show [17]. A translucent denture base material results in a natural-looking appearance due to the “chameleon” effect, which allows the adjacent and underlying structures to reflect or show through [18].

Three-dimensional printing technology for denture base fabrications is considered new in the field of dentistry, and, to the authors’ knowledge, no previous study has assessed the water sorption, solubility, and translucency of 3D-printed resins. Therefore, the current study aimed to assess water sorption/solubility and translucency of 3D-printed resins in comparison to conventionally heat-polymerized acrylic resin-based materials. The null hypothesis is that there would be no significant difference between the tested materials in terms of water sorption/solubility and translucency.

## 2. Materials and Methods

### 2.1. Specimen Preparations

Sample size was computed through the power analysis, where means and standard deviations were taken from similar previously published studies [9,19]; power was set as 80% and level of significance was 0.05. Hence, the calculated sample size was 80 resin specimens (40/water sorption/solubility (µg/mm^3^), *n* = 10; 40/translucency, *n* = 10). All specimens were fabricated in accordance with the American Dental Association specification #12 for denture base polymer materials [20], with dimensions of 50 × 0.5 mm for water sorption/solubility and 15 × 2.5 mm for translucency. The conventional method was used to fabricate heat-polymerized acrylic resin (Major base 20, Major, Moncalieri, Italy), according to the manufacturer’s recommendations and a detailed method reported in a previous study [14]. After polymerization, the specimens were finished with a silicon carbide disc with sequences of 200, 400, and finally 600 grit to obtain the required dimensions using a digital caliber (0.01 µ accuracy).

**Figure 1:**
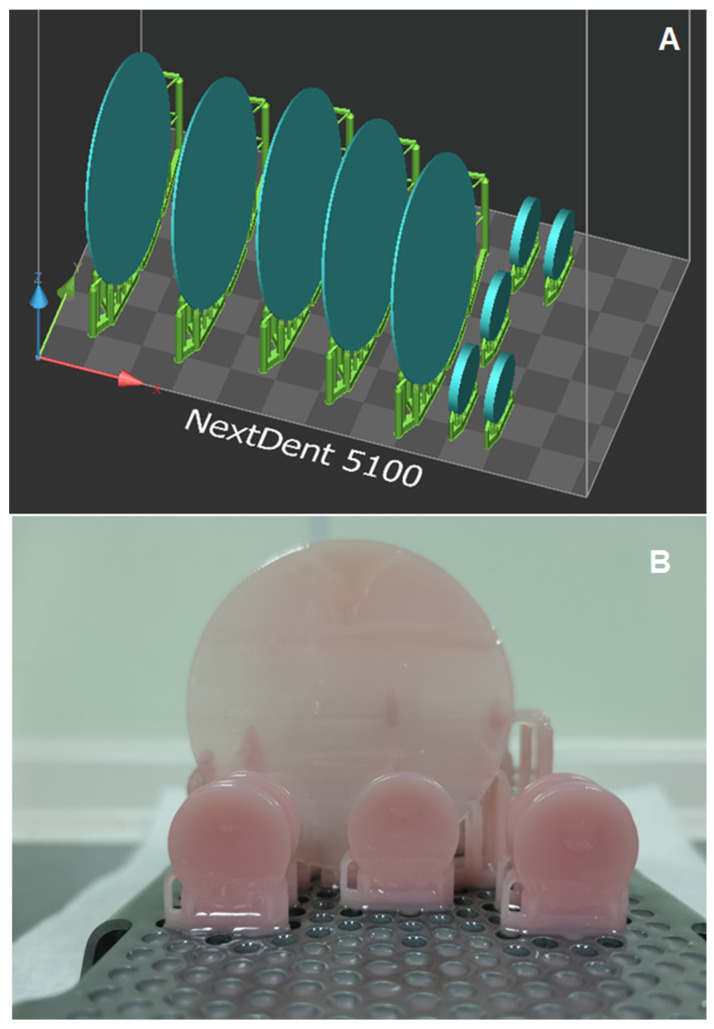
(**A**) Specimens dimension in STL file, and (**B**) 3D-printed specimens.

The dimensions of 3D-printed specimens were designed using autocued software, and then converted to an STL file for printing (Figure 1A). Three printers were used; printing was achieved by following the manufacturers’ recommendations (Figure 1B). Three commercially available 3D-printed resins were investigated: NextDent, FormLabs, and Asiga. Details regarding printing resins, printers used, and printing parameters are summarized in Table 1. As the polymerization process was completed for printed specimens, the specimens were finished using the previously mentioned method for the control specimens, thereby creating similar dimensions amongst all tested specimens. Thus, specimens with improper dimensions were excluded from the study. Finishing of all specimens was completed by rinsing them with water, and any debris or contaminations were removed using an ultrasonic cleaner, before they were eventually stored in distilled water at 37 °C.

### 2.2. Water Sorption/Solubility and Translucency Measurements [T1]

Water solubility/sorption tests were conducted according to the recommended ISO 20795-1:2013 standards [21]. Each specimen’s volume (V) was determined by obtaining the average values of the diameter and thickness. The specimen’s diameter was measured at three points, and the average of those points was recorded as the specimen’s diameter. The specimen’s thickness was measured at five points; the first point was at the center, and the other four points were on the outlines. A desiccator containing dried silica gel was used to store the specimens for 23 h at 37 °C (±1 °C). All the specimens were then stored in a second desiccator for 1 h at 23 °C (±2 °C). The specimens were then weighed using a scale (accuracy of 0.1 mg). The desiccation cycle was continuous until a constant mass (m1) was obtained. The dried specimens were submerged in water for 14 days at a temperature of 37 °C (±1 °C) [22]. After removing the specimens, they were left outside for 60 min and dried with a clean towel, before being weighed again (m2). To obtain the water sorption value (µg/mm^3^), the following formula was used: (Wsp = (m2 − m1)/specimen volume (V)). Once the specimens were weighed for sorption, they were dried to achieve a constant mass (m3); the drying process was similar to the method used for m1 determination. The solubility values of the specimens were obtained using the following formula: (Wsl = (m3 − m1)/specimen volume (V)) [12,13].

### 2.3. Translucency Measurements

A spectrophotometer was used to measure the reflectance values (Color-Eye^®^ 7000A, X-Rite, Carlstadt, NJ, USA). As per the manufacturer’s recommendations, a black trap and white tile were used to calibrate the spectrophotometer. A port was used to stabilize the specimens, and the white or black reference material was supported at the back of the specimens to keep the arm closed. The color measurements of the coordinates (L*, a*, b*) using the Commission Internationale de l’Eclairage system were obtained for the discs against the white and black backgrounds, and the system automatically provided the average. The tabulated data were used to calculate the translucency (TR) ratio, and the following equation was used to obtain the results:

TR = [(Lwhite* − Lblack*)^2^ + (awhite* − ablack*)^2^ + (bwhite* − bblack*)^2^]^1/2^. (1)

### 2.4. Thermal Cycling Effect and Repeated Measures (T2)

**Figure 2:**
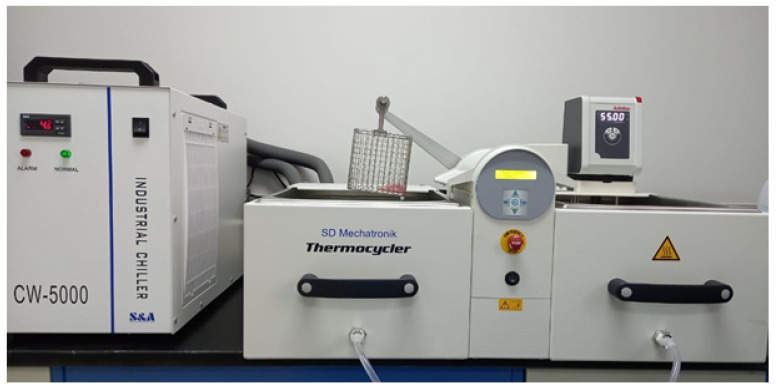
Specimen aging using thermocycling machine.

The specimens underwent 5000 thermal cycles (TCs) (Figure 2). Cycling was performed in distilled water at 5 °C with a 30 s dwell time and at 55 °C for a similar dwell time. The transfer time between both temperatures was 5 s. The 5000 TCs replicated a time frame of 6 months in the oral cavity [1,23,24]. The mean value for volume was calculated to establish a baseline that corresponded to the weight’s mean value (5000 TCs then dried in silica gel). The following formulas were used for water sorption and solubility, respectively: (Wsp 5000 TC = m4 − m1/specimen volume (V)) and (Wsl 5000 TC = m5 − m1/specimen volume (V)).

In the present study, m1 was the dry specimen’s mass prior to water storage in mg, m4 was the specimen’s mass after completing 5000 TCs in mg, m5 was the specimen’s mass once it was redried in mg, and V was the specimen’s volume in mm^3^ [9]. All specimens underwent the translucency test again after thermal cycling, to compare the impact of this treatment on the materials’ translucency values.

### 2.5. Statistical Analysis

The SPSS v.23 social sciences statistical package was used to analyze the data. Data normality was tested first using the Shapiro–Wilk test, and insignificant *p*-values proved that the data were normally distributed. Hence, parametric tests were used for the inferential statistical analysis. One-way ANOVA was used to determine the relationship between the types of materials used and the tested properties. A post hoc test was employed for pairwise comparisons. Two-way ANOVA was used to study the combined effect of type of materials and thermocycling effects on the tested properties. A two-independent-sample *t*-test was used to evaluate the effect of thermal cycling, and the outcomes were compared across materials. A *p*-value less than 0.05 was considered statistically significant.

## 3. Results

**Figure 3:**
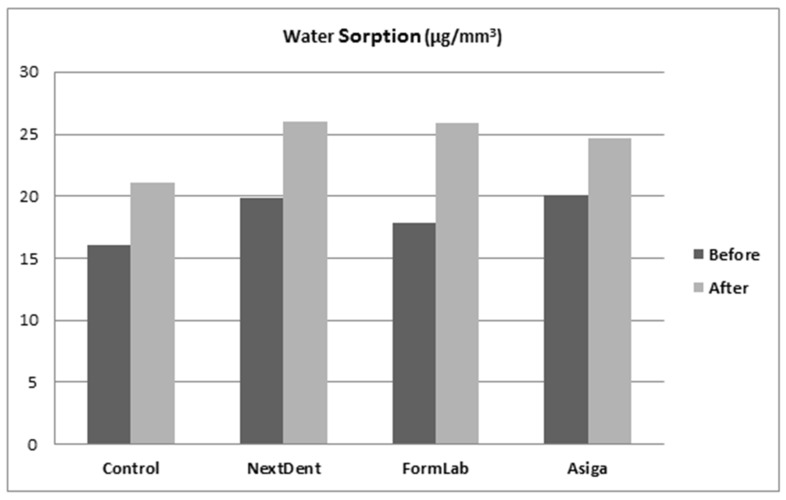
Water sorption values before and after thermal cycling.

**Figure 4:**
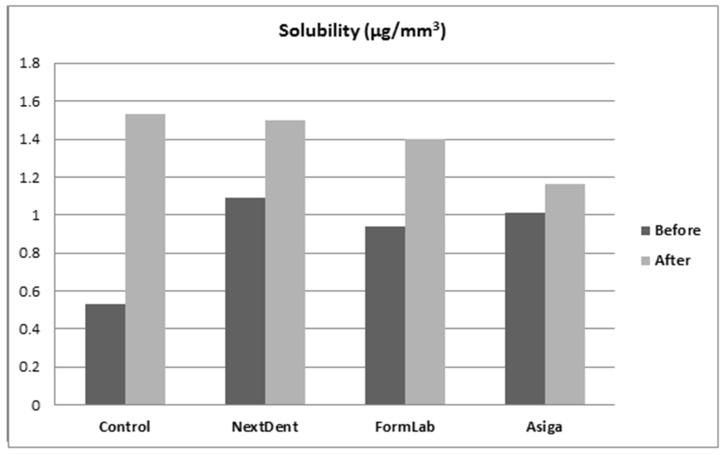
Solubility values before and after thermal cycling.

**Figure 5:**
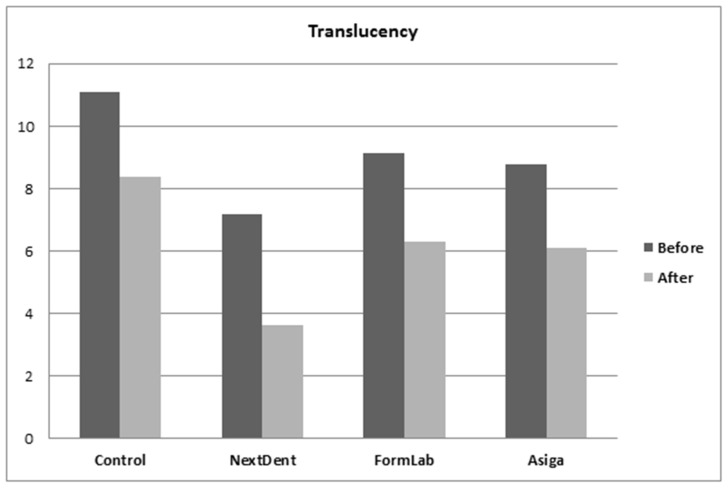
Translucency values before and after thermal cycling.

The mean and standard deviation (SD) results from the ANOVA post hoc Tukey tests for water sorption/solubility and translucency are summarized in Table 2 and Figure 3, Figure 4 and Figure 5. Variations in water sorption due to changes in the materials were found to be statistically significant before and after thermal cycling (*p* < 0.001). The control group showed the lowest water sorption value in comparison to 3D-printed resins (*p* < 0.001). Among the 3D-printed resins, FormLabs showed the lowest water sorption when compared with Asiga and NextDent. In terms of thermal cycling effect per material, the water sorption was significantly increased (*p* < 0.001). The combined effect of thermal cycling and the type of material on water sorption was also found to be statistically significant (*p* < 0.001) (Table 3).

One-way ANOVA was used to test the effect of material type on its solubility. It was found to be statistically significant with or without thermal cycling (*p* < 0.001). Before thermal cycling, 3D-printed resins showed significantly increased solubility in comparison to the control group (*p* < 0.001). After thermal cycling, Asiga showed a significant decrease in comparison to the control and other 3D-printed groups (*p* < 0.001). No significant differences were found between the control and the NextDent and FormLabs resins (*p* > 0.05). When comparing the thermal cycling effect for each material, the solubility was significantly increased (*p* < 0.001). Furthermore, the combined effect of thermal cycling and the type of material on water sorption was found to be statistically significant (*p* < 0.001) (Table 3).

Translucency with or without the thermal cycling effect was found to be highest in the control group (*p* < 0.001). Among 3D-printed resins, NextDent showed the lowest translucency values in comparison to FormLabs and Asiga (*p* < 0.001), while no significant difference was found between FormLabs and Asiga with or without thermal cycling (*p* = 0.824, *p* = 0.932, respectively). Thermal cycling decreased the translucency of all tested materials (*p* < 0.001). Furthermore, two-way ANOVA revealed that the combined effect of materials and thermal cycling was statistically insignificant (*p* = 0.324) (Table 3).

## 4. Discussion

The current study aimed to assess and compare the water sorption/solubility and translucency of denture base resins (NextDent, FormLabs, and Asiga 3D-printed resins with a heat-polymerized resin as the control), and to determine whether thermal cycling had any effect on these properties. The null hypothesis, that there would be no difference between the tested materials in terms of water sorption/solubility and translucency, was rejected because all the tested properties were significantly affected.

Acrylic resins have a tendency to absorb water or solvents gradually over long periods of time, principally because of the inherent nature of resin molecules and the resin’s polarity properties [11,25]. Water uptake at high equilibrium levels softens acrylic-based resins, leading to chemical degradation and residual monomer elution [25]. The increased water sorption by denture base resins creates internal stresses and crack formation over time, ultimately affecting the mechanical properties, especially the color stability of dentures [26]. Water sorption and solubility are used to assess the acrylic resins’ ability to resist surrounding oral fluids and are indicators of the denture base resins’ durability [27]. Therefore, water sorption/solubility must be kept as low as possible.

According to the current study’s findings, the water sorption of 3D-printed resins increased in comparison to the heat-polymerized resin. This finding is in agreement with Perea-Lowery [22] and Berli et al. [9], who found higher water sorption values for 3D-printed resins. The increase in water sorption may be due to the polymerization technique. Low polymerization degrees of 3D-printed resins resulted in unreacted monomers [1,22]. In addition to other constituents of polymerized resins, such as crosslinking agents, plasticizers, initiators, or soluble materials, this leads to high water sorption [28]. Another factor that affects water sorption is the differences in chemical composition between heat-polymerized resins and 3D-printed resins [12,22,27].

In addition to the polymerization technique, the printed layering technique used to manufacture 3D-printed resins might be a reason for the increased amount of water sorption. The absorbed water enters between the layers, and then the water molecules are diffused into the resin’s polymer, which allows interpolymeric spaces to be filled with water, forcing the polymer chain away from other chains [1,29,30]. This phenomenon might adversely affect the layering interface, which leads to swelling of the resin and separates the printed layers [13,26]. The presence of voids in 3D-printed resins was confirmed with scanning electron microscopy [1]. Absorbed water diffuses and penetrates the empty spaces and voids, which could explain the increased levels of water sorption in 3D-printed resins [22].

Thermal cycling was applied to simulate clinical use; it is considered to be a guideline for material behavior in the oral cavity. Moreover, increased temperature has been shown to accelerate water uptake [29]. Therefore, all resin materials showed increased water sorption after thermal cycling, and 3D-printed materials exhibited the same behavior [1].

The quantity of water-soluble elements, unreacted monomers, plasticizers, and initiators that leached out while the specimens were immersed in water symbolizes the solubility of acrylic resins [31]. Lassila and Vallittu [32] reported a correlation between the water sorption/solubility of polymers and stated that materials with high solubility will absorb more water. Furthermore, there is a direct correlation between solubility and mechanical properties, whereby an increase in the solubility of denture base resin leads to a decrease in the mechanical properties [33]; therefore, the solubility must also be low.

The results of the present study showed that the solubility of 3D-printed resins increased in comparison to the heat-polymerized resin. This finding is similar to a previous study reporting that the solubility of 3D-printed acrylic resins increased in comparison to pressed resin for occlusal devices [9]. The increased solubility is due to the leaching out of the residual monomers in addition to other soluble substances [22,27,29]. After thermal cycling, the solubility of the NextDent resin and the control was similar, while the solubility levels of FormLabs and Asiga increased, indicating greater loss of substances. This loss may be due to leakage or to the fact that the water uptake was significantly high, eventually resulting in the water permanently binding to the material [9,22].

According to ISO 20795, during the storage of the specimens, 32 µg/mm^3^ (water sorption) is the acceptable volumetric mass increase in denture base materials per volume, and the value of the soluble substances must be 1.6 µg/mm^3^ or less. After calculation for each value, the probability average for water sorption (1.0) was found to be lower than ISO. Moreover, the probability value was the same for solubility before the thermal cycling effect but dropped to 0.8 after thermal cycling. All water sorption/solubility values of 3D-printed resins were lower than the ISO recommendation for maximum water sorption; hence, the results for the three 3D-printed resins demonstrate that they are suitable for clinical application. However, further investigations are required to interpret the variations in water sorption/solubility between 3D-printed materials with different aging effects.

The aesthetic demands of edentulous patients must be considered, and the resins that are used must result in optimum levels of satisfaction. Therefore, clinicians are advised to cautiously deliberate between using superior materials that exhibit better optical properties while also considering their stability over time. Translucency is regulated by the interaction of light that undergoes transmission, absorption, reflection, and scattering in a substance [34]. Very few studies have investigated the color stability of 3D-printed denture base resins with different staining solutions, and no trial has been conducted to evaluate and compare the translucent properties of 3D-printed resins.

In relation to our study, there was a significant difference between the control group and three 3D-printed resin materials, showing a significant decrease in translucency. The decrease in translucency could be due to the effect of water sorption, as described in previous studies [14,35,36], which reported that absorbed water diffuses into a polymer network resulting in hydrolysis and acrylic zones packed with water masses. These water masses could disrupt the ultraviolet beam passage, decreasing the material’s translucency [35].

Moreover, 3D-printed resins contain fillers, and these fillers have different refractive indices [1,14]. According to Lee et al. [34], the value of translucency decreased as the amount of filler increased. Shin et al. [37] observed that water storage for a month resulted in water sorption, which could affect the optical properties of 3D-printed resins. Kim et al. [38] conducted a study that assessed the translucency and color stability of 3D-printed provisional resins; they found minor changes in translucency and noted that 3D-printed dental prostheses had a yellowish color while their opacity increased after water storage for 6 months [38].

To be clinically relevant, 3D-printed resins are deemed suitable for clinical applications when their water sorption/solubility values adhere to the ISO recommendations. However, the aesthetics of 3D-printed denture base resins are still questionable, and further investigations are needed to overcome the opacity resulting from the changes in printing parameters, which could affect the physical properties of printed resins, such as printing orientations, printing layer thickness, light intensity, post-curing time and temperatures, and/or printed resin modifications [5,39,40,41,42]. It was reported that the outcomes of printed objects vary with different printing orientations. Printing at a 0° orientation results in fewer layers per specimen, and this contributes to improving their physical properties and flexural properties, as the load direction is perpendicular to printing layers [5]. Due to the outcome variations with printing orientations, the printing orientation should be carefully decided to fabricate products with appropriate properties. Additionally, the printing layer thickness affects the properties of 3D-printed resin, whereby a decrease in thickness increases the degree of conversion [40]. As the degree of polymerization increases, the amount of uncured monomer decreases, subsequently improving the 3D-printed resin properties [41]. Other factors that could improve the degree of polymerization are post-curing time and temperature. The physico-mechanical properties of 3D printed resins increase with the post-curing time and temperature [41,42]. Therefore, in order to enhance the properties of 3D-printed resins, it is recommended that their printing parameters be altered to produce optimum printed prostheses.

The strength of this study was that it investigated the water sorption/solubility and translucency of different 3D-printed resins, as well as the thermal cycling effect on these properties. However, the study had some limitations. Firstly, the presence of oral fluid, saliva pH, and enzymes, which may result in further degradation of resin materials, was not investigated. Furthermore, fluids that create stains, as well as denture cleansing solutions, may affect the water sorption, solubility, and translucency of resins. Further investigations of the effect of oral fluids and/or using denture cleansers and beverages on the water sorption/solubility and translucency of 3D-printed resins are recommended.

## 5. Conclusions

The three investigated 3D-printed denture base resins had high water sorption and solubility values. The translucency of the 3D-printed resins was low in comparison to the heat-polymerized resin control before thermal cycling; however, the translucency of NextDent was comparable to that of the heat-polymerized denture base resin.

## Figures and Tables

**Table 1:** Materials and equipment used in the study.

Material Brand Name/Printers/Printing Technology	Composition	Printing Parameters			Post Printing Conditions		
		Layer Thickness	Orientations	Light Source/Intensity	Rinsing/ Cleaning	Post Curing Machine	Post Curing Time/Temperature
NextDent Denture 3D+/ NextDent 5100 3D NextDent B.V/ Stereolithography	Methacrylic oligomers, methacrylate monomer, inorganic filler, phosphine oxides, pigments	50 µm	90°	Blue UV-A 405 nm	Isopropyl alcohol 99.9%,	LC-D Print Box, 3D systems, Vertex Dental B.V., Soesterberg, Netherland	10 min/60 °C
Denture base OP resin, FormLabs/ Form 3+ Formlabs/ Stereolithography	Biocompatible photopolymer resin	50 µm	90°	UV laser 405 nm, 120 mW	Isopropyl alcohol 99.9%,	FormCure (Formlabs Form Cure)	15 min/60 °C
*ASIGA* DentaBASE, *ASIGA* MAX UV/ *ASIGA*, Erfurt, Germany/ Digital light processing (DLP)	Polymer	50 µm	90°	UV LED (385 nm–405 nm	Isopropyl alcohol 99.9%,	Asiga Flash UV Curing Chamber	15 min/80 °C

**Table 2:** Mean, standard deviation (SD), and significances of materials for all tested properties.

Property	Thermal Cycling Effect	Materials Mean (SD)				ANOVA *F-* and *p*-Values
		Control	NextDent	FormLabs	Asiga	
Water Sorption (µg/mm^3^)	Before	16.1(1.1)	19.83(1.2) ^a^	17.86(0.83)	20.1(1.2) ^a^	*F* = 29.262 *p* = 0.000 *
	After	21.1(1.3)	25.99(1.3) ^a^	25.92(1.2) ^a^	24.64(0.98) ^a^	*F* = 38.015 *p* = 0.000 *
	*p* value	0.000 *	0.000 *	0.000 *	0.000 *	
Solubility (µg/mm^3^)	Before	0.53(0.1)	1.09(0.2) ^a^	0.94(0.12) ^a^	1.01(0.1) ^a^	*F* = 30.149 *p* = 0.000 *
	After	1.53(0.07) ^a^	1.5(0.2) ^a^	1.4(0.24) ^a^	1.16(0.14)	*F* = 10.274 *p* = 0.000 *
	*p* value	0.000 *	0.000 *	0.000 *	0.017 *	
Translucency	Before	11.09(1.1)	7.18(0.87)	9.14(0.98) ^a^	8.78(0.74) ^a^	*F* = 29.326 *p* = 0.000 *
	After	8.38(0.96)	3.62(0.74)	6.29(0.71) ^a^	6.09(0.5) ^a^	*F* = 68.002 *p* = 0.000 *
	*p* value	0.000 *	0.000 *	0.000 *	0.000 *	

* Statistically significant at the 0.05 level. The same small letter “^a^” in each row denotes a statistically insignificant difference between the means.

**Table 3:** Effect of thermocycling (before and after) and type of material on tested properties using two-way ANOVA.

Properties	Variables	Type III Sum of Squares	df	Mean Square	*F*-Value	*p*-Value
Water Sorption (µg/mm^3^)	Materials	229.884	3	76.628	58.555	0.000 *
	Thermocycling effect	707.455	1	707.455	540.600	0.000 *
	Materials × thermocycling	36.850	3	12.283	9.386	0.000 *
	Error	94.223	72	1.309		
	Total	37,813.148	80			
Solubility (µg/mm^3^)	Materials	0.805	3	0.268	11.072	0.000 *
	Thermocycling effect	5.050	1	5.050	208.482	0.000 *
	Materials × thermocycling	1.939	3	0.646	26.681	0.000 *
	Error	1.744	72	0.024		
	Total	113.962	80			
Translucency	Materials	188.669	3	62.890	87.503	0.000 *
	Thermocycling effect	174.434	1	174.434	242.701	0.000 *
	Materials × thermocycling	2.540	3	0.847	1.178	0.324
	Error	51.748	72	0.719		
	Total	5006.175	80			

* Statistically significant at the 0.05 level.

## Data Availability

Not applicable.

## References

[1] Gad M.M., Fouda S.M., Abualsaud R., Alshahrani F.A., Al-Thobity A.M., Khan S.Q., Akhtar S., Ateeq I.S., Helal M.A., Al-Harbi F.A. (2021). Strength and Surface Properties of a 3D-Printed Denture Base Polymer. J. Prosthodont..

[2] Prpić V., Schauperl Z., Ćatić A., Dulčić N., Čimić S. (2020). Comparison of Mechanical Properties of 3D-Printed, CAD/CAM, and Conventional Denture Base Materials. J. Prosthodont..

[3] Chhabra M., Nanditha Kumar M., Raghavendra Swamy K.N., Thippeswamy H.M. (2022). Flexural strength and impact strength of heat-cured acrylic and 3D printed denture base resins—A comparative in vitro study. J. Oral Biol. Craniofac. Res..

[4] Al-Dwairi Z.N., Tahboub K.Y., Baba N.Z., Goodacre C.J., Özcan M. (2019). A comparison of the surface properties of CAD/CAM and conventional polymethylmethacrylate (PMMA). J. Prosthodont..

[5] Shim J.S., Kim J.E., Jeong S.H., Choi Y.J., Ryu J.J. (2020). Printing accuracy, mechanical properties, surface characteristics, and microbial adhesion of 3D-printed resins with various printing orientations. J. Prosthet. Dent..

[6] Bidra A.S., Taylor T.D., Agar J.R. (2013). Computer-aided technology for fabricating complete dentures: Systematic review of historical background, current status, and future perspectives. J. Prosthet. Dent..

[7] Alp G., Murat S., Yilmaz B. (2019). Comparison of flexural strength of different CAD/CAM PMMA-based polymers. J. Prosthodont..

[8] Figuerôa R.M.S., Conterno B., Arrais C.A.G., Sugio C.Y.C., Urban V.M., Neppelenbroek K.H. (2018). Porosity, water sorption and solubility of denture base acrylic resins polymerized conventionally or in microwave. J. Appl. Oral. Sci..

[9] Berli C., Thieringer F.M., Sharma N., Müller J.A., Dedem P., Fischer J., Rohr N. (2020). Comparing the mechanical properties of pressed, milled, and 3D-printed resins for occlusal devices. J. Prosthet. Dent..

[10] Machado C., Rizzatti-Barbosa C.M., Gabriotti M.N., Joia F.A., Ribeiro M.C., Sousa R.L. (2004). Influence of mechanical and chemical polishing in the solubility of acrylic resins polymerized by microwave irradiation and conventional water bath. Dent. Mater..

[11] Saini R., Kotian R., Madhyastha P., Srikant N. (2016). Comparative study of sorption and solubility of heat-cure and self-cure acrylic resins in different solutions. Indian J. Dent. Res..

[12] Pfeiffer P., Rosenbauer E.U. (2004). Residual methyl methacrylate monomer, water sorption, and water solubility of hypoallergenic denture base materials. J. Prosthet. Dent..

[13] Hada T., Kanazawa M., Iwaki M., Katheng A., Minakuchi S. (2021). Comparison of Mechanical Properties of PMMA Disks for Digitally Designed Dentures. Polymers.

[14] Gad M.M., Abualsaud R., Alqarawi F.K., Emam A.M., Khan S.Q., Akhtar S., Mahrous A.A., Al-Harbi F.A. (2021). Translucency of nanoparticle-reinforced PMMA denture base material: An in-vitro comparative study. Dent. Mater. J..

[15] Dayan C., Guven M.C., Gencel B., Bural C.A. (2019). A Comparison of the Color Stability of Conventional and CAD/CAM Polymethyl Methacrylate Denture Base Materials. Acta. Stomatol. Croat..

[16] Waliszewski M. (2005). Restoring dentate appearance: A literature review for modern complete denture esthetics. J. Prosthet. Dent..

[17] Johnston W.M., Ma T., Kienle B.H. (1995). Translucency parameter of colorants for maxillofacial prostheses. Int. J. Prosthodont..

[18] Ryan E.A., Tam L.E., McComb D. (2010). Comparative translucency of esthetic composite resin restorative materials. J. Can. Dent. Assoc..

[19] Alfouzan A., Alotiabi H.M., Labban N., Al-Otaibi H.N., Al Taweel S.M., AlShehri H.A. (2021). Color stability of 3D-printed denture resins: Effect of aging, mechanical brushing and immersion in staining medium. J. Adv. Prosthodont..

[20] American Dental Association (1975). Revised American Dental Association specification no. 12 for denture base polymers. J. Am. Dent. Assoc..

[21] International Organization for Standardization (ISO) (2013). Dentistry—Base Polymers—Part 1: Denture Base Polymers; ISO 20795-1:2013(en).

[22] Perea-Lowery L., Gibreel M., Vallittu P.K., Lassila L.V. (2021). 3D-Printed vs. Heat-Polymerizing and Autopolymerizing Denture Base Acrylic Resins. Materials.

[23] Gale M.S., Darvell B.W. (1999). Thermal cycling procedures for laboratory testing of dental restorations. J. Dent..

[24] Jadhav V., Deshpande S., Radke U., Mahale H., Patil P.G. (2021). Comparative evaluation of three types of denture base materials with saliva substitute before and after thermocycling: An in vitro study. J. Prosthet. Dent..

[25] Barsby M.J. (1992). A denture base resin with low water absorption. J. Dent..

[26] Iwaki M., Kanazawa M., Arakida T., Minakuchi S. (2020). Mechanical properties of a polymethyl methacrylate block for CAD/CAM dentures. J. Oral Sci..

[27] Arima T., Murata H., Hamad T. (1996). The effects of cross-linking agents on the water sorption and solubility characteristics of denture base resin. J. Oral Rehabil..

[28] Craig R.G., Powers J.M., Sakaguchi R.L. (2011). Craig’s Restorative Dental Materials.

[29] Lin C.T., Lee S.Y., Tsai T.Y., Dong D.R., Shih Y.H. (2000). Degradation of repaired denture base material in simulated oral fluid. J. Oral Rehabil..

[30] Vallittu P.K., Ruyter I.E. (1997). The swelling phenomenon of acrylic resin polymer teeth at the interface with denture base polymers. J. Prosthet. Dent..

[31] Cucci A.L.M., Vergani C.E., Giampaolo E.T., Afonso M.C. (1998). Water sorption, solubility, and bond strength of two autopolymerizing acrylic resins and one heat-polymerizing acrylic resin. J. Prosthet. Dent..

[32] Lassila L.V., Vallittu P.K. (2001). Denture base polymer Alldent Sinomer: Mechanical properties, water sorption and release of residual compounds. J. Oral Rehabil..

[33] Takahashi Y., Chai J., Kawaguchi M. (1998). Effect of water sorption on the resistance to plastic deformation of a denture base material relined with four different denture reline materials. Int. J. Prosthodont..

[34] Lee Y.K. (2007). Influence of scattering/absorption characteristics on the color of resin composites. Dent. Mater..

[35] Ferracane J.L. (2006). Hygroscopic and hydrolytic effects in dental polymer networks. Dental Mater..

[36] Gad M.M., Abualsaud R., Fouda S.M., Rahoma A., Al-Thobity A.M., Khan S.Q., Akhtar S., Al-Abidi K.S., Ali M.S., Al-Harbi F.A. (2021). Color Stability and Surface Properties of PMMA/ZrO2 Nanocomposite Denture Base Material after Using Denture Cleanser. Int. J. Biomater..

[37] Shin J.W., Kim J.E., Choi Y.J., Shin S.H., Nam N.E., Shim J.S., Lee K.W. (2020). Evaluation of the Color Stability of 3D-Printed Crown and Bridge Materials against Various Sources of Discoloration: An In Vitro Study. Materials.

[38] Kim J.E., Choi W.H., Lee D., Shin Y., Park S.H., Roh B.D., Kim D. (2021). Color and Translucency Stability of Three-Dimensional Printable Dental Materials for Crown and Bridge Restorations. Materials.

[39] Gad M.M., Al-Harbi F.A., Akhtar S., Fouda S.M. (2022). 3D-Printable Denture Base Resin Containing SiO_2_ Nanoparticles: An In Vitro Analysis of Mechanical and Surface Properties. J. Prosthodont..

[40] Perea-Lowery L., Gibreel M., Vallittu P.K., Lassila L. (2021). Evaluation of the mechanical properties and degree of conversion of 3D printed splint material. J. Mech. Behav. Biomed. Mater..

[41] Aati S., Akram Z., Shrestha B., Patel J., Shih B., Shearston K., Ngo H., Fawzy A. (2022). Effect of post-curing light exposure time on the physico-mechanical properties and cytotoxicity of 3D-printed denture base material. Dent Mater..

[42] Bayarsaikhan E., Lim J.H., Shin S.H., Park K.H., Park Y.B., Lee J.H., Kim J.E. (2021). Effects of Postcuring Temperature on the Mechanical Properties and Biocompatibility of Three-Dimensional Printed Dental Resin Material. Polymers.

